# Ion-acoustic waves in weakly ionized plasmas with charge-exchange collisions

**DOI:** 10.1038/s41598-023-39780-5

**Published:** 2023-08-29

**Authors:** Abdulrahman Alharbi, L. Schiesko, T. Minea

**Affiliations:** 1 King Abdulaziz City for Science and Technology (KACST), 11442 Riyadh, Saudi Arabia; 2 Laboratoire de Physique des Gaz et des Plasmas, CNRS, Université Paris-Saclay, 91405 Orsay, France; 3 CEA, IRFM, 13108 Saint-Paul-lez-Durance, France

**Keywords:** Physics, Plasma physics

## Abstract

Ion-neutral charge-exchange collisions in plasmas of laboratory, space, and astrophysical origins are fundamental to understanding wave dissipation and wave generation phenomena. This paper implements a charge-exchange collision operator in the Boltzmann–Poisson system equations for a weakly ionized plasma. When considering an electric field perturbation, the governing kinetic equations provide significant results concerning the plasma conductivity and the dielectric function, appearing in simple, sensible forms. The present analysis reveals a backward wave propagation phenomenon at maximum conductivity when the wavenumber of the plasma wave is smaller than the reciprocal of the ion-neutral collisions mean free path. In addition, it is shown that ion-neutral coupling resulting from charge-exchange collisions enhances ion-acoustic waves below and beyond the ion plasma frequency and leads to the onset of a fundamental instability that overcomes Landau damping under certain circumstances. The collisionless model is recovered as a limiting case, i.e., in the asymptotic limit of a long mean free path.

The ion-acoustic wave instability is a well-known kinetic instability that occurs in non-equilibrium plasmas and has been widely studied. In a collisionless, unmagnetized plasma with a single species of positive ion, linear kinetic theory predicts destabilization of the Landau interaction when the drift velocity between electrons and ions is greater than the phase velocity of an electrostatic wave^1^. Otherwise, the ideal case is that the longitudinal plasma waves arising due to electrostatic perturbations are damped.

In weakly ionized plasmas, collisions with neutral particles play an important role in ion-acoustic wave damping and the growth rate of instability. Vranjes and Poedts^2^ reported that ion-neutral collisions, which predominate at low temperatures, reduce the ion-acoustic wave damping rate for a low density of neutrals, and upon increasing the neutral density, the mode becomes evanescent before it reappears for a larger number of neutrals. Although this may appear counter-intuitive given that one may expect a combination of collisional and collisionless damping mechanisms, neutral pressure has an effect^3^, and whether a pressure threshold exists at which instability can be excited or suppressed depends on the collision frequency, or equivalently, on the mean free path. An experimental study^4^ has also verified that ion-neutral collisions support the ion-acoustic wave propagation for longer distances at higher neutral gas pressure. These earlier studies indicate that the dynamical behavior of neutrals in weakly ionized plasmas must be properly treated in kinetic theory to understand wave phenomena.

In general, ion-neutral charge-exchange collisions appear in a variety of physical settings, such as the interstellar medium^5^, as well as antimatter^6^ and tokamak plasmas^7^. The charge-exchange reaction is a process whereby a neutral and ion exchange identities; both particle species are locally conserved, but energy and momentum are exchanged. The influence of these collisions in partially ionized plasmas has been investigated theoretically within the fluid formalism^8,9^ and the context of plasma sheath^10^. Nevertheless, charge-exchange collisions may give rise to potential perturbations and energy losses of positive ions when colliding with neutrals, causing the Ion Velocity Distribution Function (IVDF) to become linearly unstable^11^. From this point of view, a self-consistent numerical analysis by Schiesko et al.^12^ showed that the ion-acoustic instability was triggered by a non-monotonically decreasing ion’s VDF (i.e., satisfying Penrose’s criterion^13^) inducing potential modulations. Furthermore, as demonstrated by the convective nature of this instability, no net damping was observed for the chosen parameters^12^.

The underlying mechanism leading to the onset of a particular class of ion-acoustic instabilities in connection with resonant charge exchange is yet to be understood from a theoretical perspective. The present paper aims to develop a theoretical description for interpreting such instabilities within a plasma kinetic theory framework after investigating the essential physical aspects associated with charge-exchange collisions thoroughly.

We consider a weakly ionized plasma composed of electrons, singly (positive) charged ions and a uniform gas background of cold neutrals. The problem treats the one-dimensional spatial situation for analytical convenience so that the *z*-axis is arbitrarily chosen in the direction of the wave vector ${\textbf{k}}$. The homogeneous gas of neutrals corresponds to a distribution function of the form $f_{n}\left( v \right) =n_{n}\delta \left( v \right)$; and the ions are considered at rest after having made an elastic charge exchange. Here $n_{n}$ is the constant number density of the background gas.

Starting from a stationary equilibrium with a vanishing electric field $E _{eq}\left( z \right) =0$, the system’s equilibrium state is modified by weak electric field perturbations: $E \left( z, t \right) =\delta E \left( z, t \right)$. Moreover, the equilibrium distribution function is Maxwellian:

1 $$\begin{aligned} F_{e, i}\left( v \right) =\frac{n_{eq}}{\sqrt{\pi }\ v_{T_{e, i}}}\ \textrm{exp} \left( -\frac{v^{2}}{v_{T_{e, i}}^{2}} \right) , \end{aligned}$$

where $n_{eq}=\int F_{e, i} \, dv$ is the equilibrium number density, and $v_{T_{e, i}}= \sqrt{2T_{e, i}/m_{e, i}}$ is the thermal speed expressed in terms of the mass $m_{e, i}$ and the normalized temperature $T_{e, i}$ measured in units of energy. The subscripts (*e*, *i*) refer to electrons and ions, respectively.

One chooses a $\delta f_{e, i}$ model where the full VDF $f_{e, i}$ is made of a static bulk $F_{e, i}$ and a perturbation $\delta f_{e, i}$. As such, $f_{e, i} \left( z, v, t \right) =F _{e, i}\left( v \right) +\delta f_{e, i} \left( z, v, t \right)$, with $\left| F_{e, i}\right| \gg \left| \delta f_{e, i} \right|$. *A priori*, the electrons satisfy Boltzmann’s distribution $n_e\left( z, t \right) =n_{eq} \exp \left( e\varphi \left( z, t \right) /T_{e} \right)$, having defined the elementary charge *e* and the electric potential $\varphi$. In addition, an adequate assumption to be made for the electrons is that the perturbed potential energy is small compared to $T_{e}$, i.e., $n_{e}\simeq n_{eq}+en_{eq} \varphi /T_{e}$.

Based on the above considerations, the Boltzmann equation for the evolution of the IVDF $f_{i}$ is given by:

2 $$\begin{aligned} \begin{aligned} \frac{\partial f_{i}}{\partial t}+v\frac{\partial f_{i}}{\partial z}+\frac{eE}{m_{i}}\frac{\partial f_{i}}{\partial v}={\mathscr {C}}\left( f_{i} \right) . \end{aligned} \end{aligned}$$

In Eq. (2), ${\mathscr {C}}\left( f_{i} \right)$ designates the charge-exchange operator^10,14^:

3 $$\begin{aligned} \begin{aligned} {\mathscr {C}}\left( f_{i} \right) =\delta \left( v \right) \int _{-\infty }^{\infty }dv \ \nu _{cx} \left( v \right) f_{i}-\nu _{cx} \left( v \right) f_{i}, \end{aligned} \end{aligned}$$

where the collision frequency $\nu _{cx} \left( v \right) =v/\lambda _{cx}$ is given in terms of the ion-neutral (charge-exchange) mean free path $\lambda _{cx}$, and we have assumed that neutrals and ions have the same mass. It is worth mentioning that the collision operator given by Eq. (3), which conserves the particle number density and satisfies Boltzmann’s *H-theorem* in its general form^14^, takes into account the momentum transfer between ions and neutrals, and vice versa. The Dirac delta appearing in the first term in the collision operator is due to the contribution of the defined neutrals velocity distribution function $f_{n}$. To a good approximation for weakly ionized rare gas plasmas, the mean free path is assumed to be constant in velocity^15^. Note that electron-neutral impact ionization may also be accounted for by adding a corresponding term to the operator.

The ion flux is essentially the total flux (because electrons follow the Maxwell-Boltzmann distribution), and the perturbed IVDF is related to the current density via $J=e\int _{-\infty }^{\infty }dv \ v \delta f_{i}$. The linearized Boltzmann–Poisson system describing the IVDF in response to the electric field perturbation is obtained:

4 $$\begin{aligned}&\frac{\partial \delta f_{i}}{\partial t}+v\frac{\partial \delta f_{i}}{\partial z}+\frac{eE}{m_{i}}\frac{\partial F_{i}}{\partial v}=\frac{\delta \left( v \right) }{e\lambda _{cx}}\ J-\frac{v}{\lambda _{cx}} \delta f_{i}, \end{aligned}$$

5 $$\begin{aligned}&\frac{\partial E}{\partial z}=\frac{e}{\varepsilon _{0}} \int _{-\infty }^{\infty }dv \ \delta f_{i}-\frac{\varphi }{\lambda ^{2}_{De}}, \end{aligned}$$

where $\lambda ^{2}_{De}=\varepsilon _{0}T_{e}/e^{2}n_{eq}$ is the electrons Debye length and $\varepsilon _{0}$ is the vacuum permittivity. After the standard Fourier transform, where perturbed quantities vary as $\exp \left( ikz-i\omega t \right)$, the Fourier amplitude of the perturbed ion density associated with an angular frequency $\omega$ and a real wavenumber *k* reads:

6 $$\begin{aligned} \begin{aligned} \delta n_{i}\left( k, \omega \right) = \frac{i \, J_{k, \omega }}{e \omega \lambda _{cx}} -\frac{ie}{m_{i}} \ E_{k, \omega } \int _{-\infty }^{\infty }dv \ \frac{1}{\omega -kv+i\nu _{cx}\left( v \right) }\frac{\partial F_{i}}{\partial v}. \end{aligned} \end{aligned}$$

The analysis reduces to a unified expression of the current density if Eq. (6) is substituted into the Fourier transform of Poisson’s equation (5):

7 $$\begin{aligned} \begin{aligned} J_{k,\omega }=\varepsilon _{0} \, k \lambda _{cx} \, \omega \, \varepsilon _{k, \omega } \, E_{k, \omega }. \end{aligned} \end{aligned}$$

Equation (7) provides the plasma dielectric function (PDF)

8 $$\begin{aligned} \begin{aligned} \varepsilon =\varepsilon _{k, \omega } =1+\frac{1}{k^{2} \lambda _{De}^{2}}+\frac{\omega _{pi}^{2}}{k^{2}n_{eq}}\int _{-\infty }^{\infty }dv \ \frac{k}{{\omega }-kv+i\nu _{cx}\left( v \right) }\ \frac{\partial F_{i}}{\partial v}, \end{aligned} \end{aligned}$$

where $\omega _{pi}^{2}=e^{2}n_{eq}/\varepsilon _{0}m_{i}$ is the ion plasma frequency.

One can see from Eq. (7) that the current density depends on the mean free path, which encodes the effect of neutral pressure; the neutral density is varied by changing the neutral pressure, which, in turn, allows the change in $\lambda _{cx}$. In addition, since the contribution to plasma current density due to a conductivity of the form^16^ $\sigma =\sigma _{k, \omega }=-i\varepsilon _{0} \omega \left( \varepsilon _{k, \omega }-1 \right)$ is also given by $J_{k,\omega }$, the following relations hold:

9 $$\begin{aligned}&\sigma =\frac{\varepsilon _{0} \omega k \lambda _{cx}}{1-ik \lambda _{cx}}, \end{aligned}$$

10 $$\begin{aligned}&\varepsilon =\frac{1}{1-ik \lambda _{cx}}. \end{aligned}$$

It is shown now from both Eqs. (9) and (10) that the collisionless case, which is characterized by $\sigma =i \varepsilon _{0} \omega$ and vanishing $\varepsilon$, is retrieved in the asymptotic limit $\lambda _{cx} \rightarrow + \infty$.

In order to quantify dissipation due to charge-exchange collisions (and non-zero ${\text {Re}} \left\{ \sigma \right\}$), the loss tangent defines the ratio of the real and the imaginary components of the complex PDF: $\tan \theta ={\text {Re}} \left\{ \varepsilon \right\} / {\text {Im}} \left\{ \varepsilon \right\} = 1/k \lambda _{cx}$. In view of this, there exists three possible regimes in which $k \lambda _{cx}>1$, $k \lambda _{cx}<1$ or $k \lambda _{cx}=1$. Indeed, for positive *k* and real phase velocity $v_{p} =\omega / k$, the real part of the plasma conductivity Eq. (9) reaches its maximum at a group velocity

11 $$\begin{aligned} v_{g}=\left( \frac{k^{2} \lambda ^{2}_{cx}-1}{k^{2} \lambda ^{2}_{cx}+1} \right) \, v_{p}. \end{aligned}$$

A consequence of the above relation is that $\left| v_{g} / v_{p}\right| < 1$ for $k \lambda _{cx} \ne 1$. This general situation corresponds to a dispersive medium. If $k \lambda _{cx}=1$, the group velocity is zero while the phase velocity may remain finite, thus implying a stationary wave that locally confines energy. Apart from $k \lambda _{cx}$ being larger than unity, the physical situation corresponding to $k \lambda _{cx}<1$ suggests that the rate at which a wave transports energy can be opposite to the direction of the phase velocity.

Armed with Landau’s prescription^17^ in the case of $\left| \omega -kv \right| \gg \left| \nu _{cx}\left( v \right) \right|$, the integral representation of the PDF in Eq. (8) can be separated into real and imaginary components while expressing the complex frequency as $\omega =\omega _{r}+i\gamma$, where $\omega _{r}\equiv {\text {Re}}\left\{ \omega \right\}$ and $\gamma \equiv {\text {Im}}\left\{ \omega \right\}$. As long as the imaginary part of $\omega$ is zero, the analysis results in a principal value integral, in virtue of Cauchy’s theorem, and a residue component (see, e.g.,^1,16^). Accordingly, the dispersion relation reads as follows:

12 $$\begin{aligned} \begin{aligned} {\mathscr {D}} \left( k, \omega \right) =1+\frac{1}{k^{2} \lambda _{De}^{2}}-\frac{1}{1+k^{2}\lambda _{cx}^{2}}+\frac{\omega _{pi}^{2}}{k^{2}n_{eq}}\ {\mathscr {P}} \int _{-\infty }^{\infty } dv\ \frac{k}{\omega -kv} \ \frac{\partial F_{i}}{\partial v} -i\left( \frac{k\lambda _{cx}}{1+k^{2}\lambda _{cx}^{2}}+ \frac{\pi \omega _{pi}^{2}}{k \left| k \right| n_{eq}}\ \frac{\partial F_{i}}{\partial v} \bigg |_{v=\omega /k} \right) = 0, \end{aligned} \end{aligned}$$

where ${\mathscr {P}}$ denotes the Cauchy principal value. On the other hand, one can Taylor expand ${\mathscr {D}} \left( k, \omega _{r}+i\gamma \right)$ for small frequency rate $\gamma$ of the perturbation, i.e. $\left| \gamma \right| \ll \left| \omega _{r} \right|$. At first order, and denoting ${\mathscr {D}}={\mathscr {D}}_{r}+i{\mathscr {D}}_{i}$, both $\omega _{r}$ and $\gamma$ are solutions such that

13 $$\begin{aligned} {\mathscr {D}}_{r} \left( k, \omega _{r} \right) =0, \end{aligned}$$

and

14 $$\begin{aligned} {\mathscr {D}}_{i} \left( k, \omega _{r} \right) +\gamma \ \frac{\partial \ {\mathscr {D}}_{r} \left( k, \omega \right) }{\partial \omega } \bigg |_{\omega =\omega _{r}}=0. \end{aligned}$$

Furthermore, in the limit $kv/\omega _{r} \ll 1$, one can estimate the real frequency by Taylor expanding the denominator of the integrand present in the real part of Eq. (12). Up to the second order, one may arrive at a necessary consequence of Eq. (13):

15 $$\begin{aligned} \omega _{r}^{2}=\frac{k^{2}c_{s}^{2}\left( 1+k^{2}\lambda _{cx}^{2} \right) }{\left( 1+k^{2}\lambda _{De}^{2} \right) \left( 1+k^{2}\lambda _{cx}^{2} \right) -k^{2}\lambda _{De}^{2}}, \end{aligned}$$

where $c_{s}=\lambda _{De}\omega _{pi}$ is the acoustic speed. Note that the well-known ion-acoustic dispersion relation is recovered in the long mean free path limit:

16 $$\begin{aligned} \lim _{\lambda _{cx} \rightarrow +\infty }\omega _r^{2}=\frac{k^{2}c_{s}^{2}}{1+k^{2}\lambda _{De}^{2}}. \end{aligned}$$

**Figure 1:**
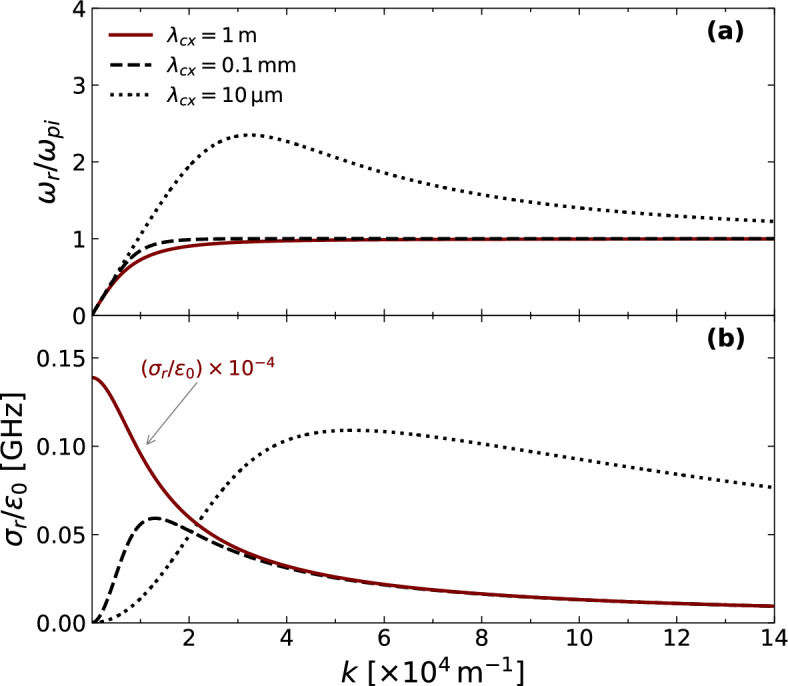
Variation of (**a**) the real frequency $\omega _{r}$ (normalized to $\omega _{pi}$) and (**b**) the real conductivity $\sigma _{r}$ (normalized to $\varepsilon _{0}$) as functions of *k* for different values of $\lambda _{cx}$. The plasma parameters chosen as a reference case in this study are $n_{eq}=10^{16} \ \mathrm {m^{-3}}$, $T_{e}=2 \ \textrm{eV}$, and $T_{i}=0.8 \ \textrm{eV}$.

Figure 1a shows the behavior of $\omega _{r}$ (normalized to the ion plasma frequency) as a function of *k* in the case of $T_{e}\gg T_{i}$. For small values of the mean free path, most notably when $\lambda _{cx}$ is on the order of 10 micrometers, the impact of charge-exchange collisions becomes significant so that $\omega _{r}$ increases beyond the ion plasma frequency for a finite *k*. In comparison with the collisionless case ($\lambda _{cx} \rightarrow +\infty$), it thus physically appears that higher neutral pressure effectively increases the ion-sound speed (i.e., the slope of the tangent line to the curve of the dispersion relation in the vicinity of $k \rightarrow 0$) of the ion-acoustic waves. Another remarkable feature is the backward wave region, where a negative slope develops at large *k*, fulfilling $k \lambda _{cx}<1$ at maximum conductivity. The effect of increasing neutral pressure can be seen as well in the plasma conductivity (see Fig. 1b), which signifies an increase from a low conductive regime (i.e., high resistivity) to one in which a maximum value of ${\text {Re}} \left\{ \sigma \right\}$ is reached, and the effect of collisions is minimized at a given *k*. When the mean free path is relatively large, the plasma remains conductive at a small *k* due to negligible charge-exchange collisions. This known variation of the conductivity in a weakly collisional plasma, such as the case for $\lambda _{cx} = 1 \, \textrm{m}$, can be seen from the red curve in Fig. 1b.

**Figure 2:**
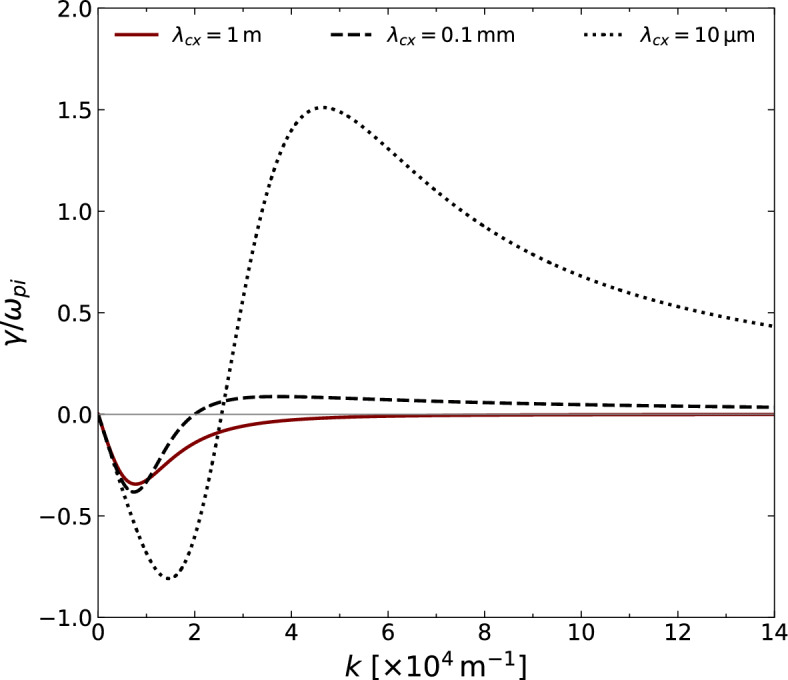
Variation of the growth rate $\gamma$ as a function of *k* for different values of $\lambda _{cx}$, and for the same parameters as in Fig. 1.

With the aid of Eqs. (12) and (14), and considering the same limit to arrive at Eq. (15), the imaginary part $\gamma$ reads:

17 $$\begin{aligned} \gamma = \frac{\omega _{r}^{3}}{2\omega _{pi}^{2}}\left( \frac{k\lambda _{cx}}{1+k^{2}\lambda _{cx}^{2}} \right) -\frac{ \sqrt{\pi } \, \omega _{r}^{4}}{k^{2} \left| k \right| v_{T_{i}}^{3}} \, \ \textrm{exp} \left( -\frac{\omega _{r}^{2}}{k^{2}v_{T_{i}}^{2}} \right) , \end{aligned}$$

where $\omega _{r}$ is given by Eq. (15). The first term in Eq. (17) is positive for a finite $\lambda _{cx}$, and it acts as an additional contribution of the plasma conductivity to that of the resonant wave-particle interaction. Although Landau’s damping rate is embodied in the second term of Eq. (17), which is negative, the value of $\lambda _{cx}$ is crucial for the sign of $\gamma$. In the collisionless limit, one may easily recover Landau’s damping rate:

18 $$\begin{aligned} \lim _{\lambda _{cx} \rightarrow +\infty } \gamma =\gamma _{Landau}= -\frac{k}{\left| k \right| }\frac{\omega _{r} \sqrt{\pi /8}}{\left( 1+k^{2}\lambda _{De}^{2}\right) ^{3/2}}\left( \frac{T_{e}}{T_{i}} \right) ^{3/2} \textrm{exp} \left( - \frac{T_{e}/2T_{i}}{ 1+k^{2}\lambda _{De}^{2}}\right) . \end{aligned}$$

In Eq. (18), $\omega _{r}$ is provided by Eq. (16).

An excitation of ion-acoustic waves ($\gamma >0$) can occur for a sufficiently small mean free path. For example, Fig. 2 shows an intermediate regime where Landau’s damping may overcome the ion-acoustic instability for large values of the mean free path (e.g., $\lambda _{cx}=1 \ \textrm{m}$). On the other hand, an extreme instability regime arises when $\lambda _{cx}$ is on the order of 10 micrometers. In comparison with Fig. 1b, one can observe that as collisions further reduce the conductivity of the plasma at long wavelengths, the Landau damping is enhanced at long and diminished at short wavelengths. The ion acoustic instability arises upon increasing the conductivity at short wavelengths.

**Figure 3:**
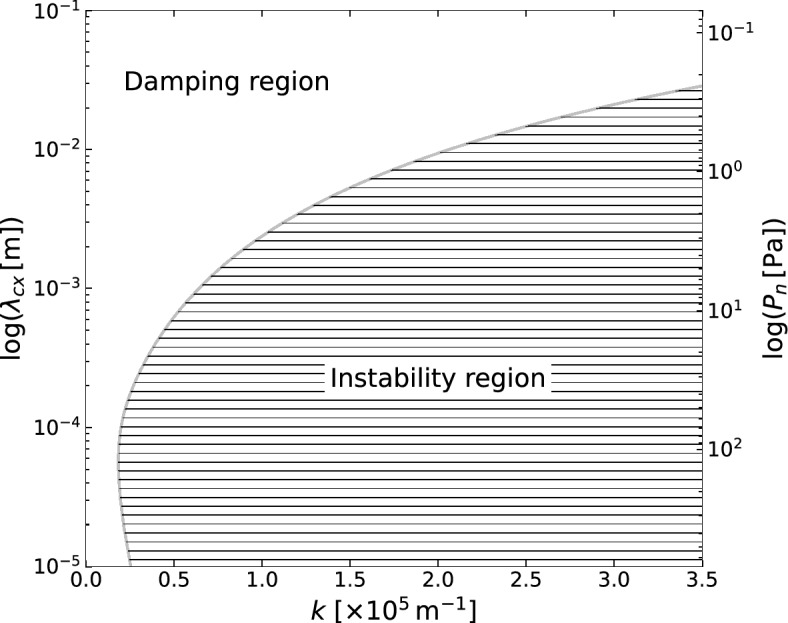
Left-hand side scale: the values of $\lambda _{cx}$ (in logarithmic scale) with respect to *k*, computed from the roots of $\gamma$ to indicate the ion-acoustic damping and instability regions. Right-hand side scale: the neutral pressure $P_{n}$ (in logarithmic scale) with respect to *k*, computed for a neutral gas at room temperature and a cross-section of $6 \times 10^{-19} \ \mathrm {m^{2}}$.

In conclusion, the linear kinetic theory allows us to explore the connection between charge-exchange collisions and essential physical properties of plasma, such as conductivity and dielectric response. Using a charge-exchange operator, the present analysis shows that the dependence of the complex dielectric and conductivity functions (Eqs. (9) and (10), respectively) upon the ion-neutral collisions mean free path is explicit. Examination of such physical properties unveils wave propagation phenomenon that manifests the interplay between the collisional process and the electric field perturbation. The relationship between the group and phase velocities demonstrates this phenomenon. It involves backward wave propagation in the case of maximum conductivity when the wavenumber of the plasma wave is smaller than the reciprocal of the mean free path.

Furthermore, charge-exchange-induced perturbations result from the coupling between ion and neutral densities via the mean free path. While the effect of charge-exchange collisions on the ion-acoustic wave corresponds to a plasma wave frequency that can exceed $\omega _{pi}$ for sufficiently small $\lambda _{cx}$, it is shown that such collisions can induce growth of the perturbations, i.e., instability. For instance, Fig. 3 depicts the mean free path over a wide range of *k* to distinguish the ion-acoustic damping region from the region of instability corresponding to the unstable root of $\gamma$. This distinction indicates the existence of a neutral pressure threshold. One may estimate as well that the range of neutral pressure that leads to instability at short wavelengths, namely for neutral gas at room temperature and a cross-section of $6 \times 10^{-19} \ \mathrm {m^{2}}$, roughly lies between 0.24 and $694 \ \textrm{Pa}$. This ideal range describes a variety of partially ionized plasmas, but one can extend the analysis to include ionizing collisions.

## Data Availability

All data used in this manuscript have been presented within the article.
